# Antibiotic Maintenance and Redevelopment of Nontuberculous Mycobacteria Pulmonary Disease after Treatment of Mycobacterium avium Complex Pulmonary Disease

**DOI:** 10.1128/spectrum.01088-22

**Published:** 2022-08-11

**Authors:** Sungmin Zo, Hojoong Kim, O. Jung Kwon, Byung Woo Jhun

**Affiliations:** a Division of Pulmonary and Critical Care Medicine, Department of Medicine, Samsung Medical Center, Sungkyunkwan University School of Medicine, Seoul, Republic of Korea; University of Guelph

**Keywords:** nontuberculous mycobacteria, antibiotics, maintenance, severity

## Abstract

Limited data are available regarding the impact of the antibiotic maintenance period on the redevelopment of nontuberculous mycobacteria-pulmonary disease (NTM-PD) after microbiological cure of Mycobacterium avium complex (MAC)-PD. This retrospective study included 631 MAC-PD patients who achieved microbiological cure between 1994 and 2021. Data on the antibiotic maintenance period, defined as the time between culture conversion and treatment completion, were collected. Redevelopment, the subsequent diagnosis of NTM-PD regardless of causative organism after microbiological cure, was investigated. Factors associated with redevelopment were analyzed after adjusting for disease severity using the body mass index, age, cavity, erythrocyte sedimentation rate, and sex (BACES) scoring system. In total, 205 (33%) patients experienced redevelopment, with a median maintenance period after culture conversion of 15.0 months (interquartile range, 13.0 to 22.0 months). A greater proportion of patients with the nodular bronchiectatic form of MAC-PD (87% versus 80%, *P* = 0.033) and a longer maintenance period (median 15.0 versus 14.0 months, *P* < 0.001) were noted in the redevelopment group compared with the nonredevelopment group. The cumulative rate of redevelopment according to the maintenance period did not differ between the >12-month and ≤12-month groups in the total patient population or the subgroups sorted according to BACES severity. No association between a maintenance period >12 months and redevelopment was identified in multivariate models. Extending the antibiotic maintenance period more than 12 months did not reduce the redevelopment rate even with adjustment for disease severity, suggesting the need to further optimize the duration of the antibiotic maintenance period.

**IMPORTANCE** Limited data are available regarding the impact of the antibiotic maintenance period on the redevelopment of Mycobacterium avium complex-pulmonary (MAC-PD) disease after microbiological cure. To improve treatment outcomes and reduce the recurrence rate, current guidelines recommend maintenance of antibiotics for a minimum of 12 months after achievement of negative culture conversion. However, the optimal duration of antibiotic therapy for MAC-PD is not currently known. Moreover, in real-world clinical practice, total antibiotic duration is mainly impacted by the length of the maintenance period; however, it is unknown whether extending the maintenance period is beneficial for preventing redevelopment of NTM-PD. Our study may help to address concerns regarding the antibiotic maintenance period after achievement of negative culture conversion in patients with MAC-PD.

## INTRODUCTION

Nontuberculous mycobacteria (NTM) are emerging worldwide as important causes of chronic pulmonary disease (PD) (1). Among more than 190 known NTM species, Mycobacterium avium complex (MAC) species most commonly cause NTM-PD (2, 3). For treatment of MAC-PD, guidelines recommend long-term, macrolide-based, multidrug therapy (4–6). However, because there is a lack of effective regimens against NTM species and adverse effects are associated with long-term use of antibiotics, the treatment success rate of MAC-PD is only 60–80% (7, 8). In addition, it is not uncommon for clinicians to encounter events of repeated recurrence during follow-up, contributing to the socioeconomic burden of NTM-PD (9–12). Thus, to improve treatment outcomes and reduce the recurrence rate, current guidelines recommend maintenance of antibiotic therapy for a minimum of 12 months after achievement of negative culture conversion (4, 6).

However, the optimal duration of antibiotic therapy for MAC-PD is not currently known (13–15). In real-world clinical practice, total antibiotic duration is mainly impacted by the length of the maintenance period; however, it is unknown whether extending the maintenance period is beneficial for preventing relapse or reinfection. Furthermore, because MAC-PD has a heterogeneous clinical course that is influenced by the phenotype, i.e., the fibrocavitary (FC) or nodular bronchiectatic (NB) form of the disease (16), and by the extent of disease (17), indiscriminate adjustment of the maintenance period may be unreasonable. Thus, optimal strategies that customize the antibiotic maintenance period based on a patient’s clinical status are needed.

A Korean research consortium recently developed a severity scoring system, referred to as the BACES system (body mass index, age, cavity, erythrocyte sedimentation rate, and sex), to predict NTM-PD outcome (18). The present study aimed to evaluate whether the maintenance period affects disease redevelopment in patients with MAC-PD who achieved microbiological cure, after adjustment for disease severity. Our study may help to address concerns regarding the antibiotic maintenance period after achievement of negative culture conversion in patients with MAC-PD.

## RESULTS

### Baseline characteristics of study patients.

The characteristics of the 631 study patients at the time of diagnosis are shown in Table 1. Approximately one-quarter (24%) of the patients had a low body mass index, and one-fifth (20%) were aged ≥65 years. Thirty-five patients had a lung cavity, and 33% were men. BACES severity assessments indicated that 41% had mild disease, 50% had moderate disease, and 9% had severe disease. The most common underlying disease was previous pulmonary tuberculosis (36%), followed by chronic obstructive pulmonary disease (12%). More than half (51%) of the patients had M. avium-PD. Most patients (82%) had the NB form, and the remaining patients (18%) had the FC form.

**TABLE 1:** Characteristics of study patients at the time of diagnosis with MAC-PD^*a*^

Characteristics	Total (*n* = 631)	Without redevelopment (*n* = 426)	With redevelopment (*n* = 205)	*P* value
Body mass index, <18.5 kg/m^2^	151 (24)	98 (23)	53 (26)	0.493
Age ≥65 yrs	123 (20)	91 (21)	32 (16)	0.109
Cavity	220 (35)	161 (38)	59 (29)	0.033
Elevated erythrocyte sedimentation rate	453 (72)	311 (73)	142 (69)	0.378
Sex, male	207 (33)	141 (33)	66 (32)	0.892
BACES severity				0.079
Mild	262 (41)	168 (39)	94 (46)	
Moderate	315 (50)	215 (51)	100 (49)	
Severe	54 (9)	43 (10)	11 (5)	
Comorbidity				
Previous pulmonary tuberculosis	225 (36)	144 (34)	81 (40)	0.189
Chronic obstructive pulmonary disease	73 (12)	49 (12)	24 (12)	>0.999
Chronic pulmonary aspergillosis	12 (2)	9 (2)	3 (2)	0.804
Lung cancer	13 (2)	9 (2)	4 (2)	>0.999
Ever-smoker^*b*^	152 (24)	103 (24)	49 (24)	>0.999
Acid-fast bacillus smear positivity	255 (40)	162 (38)	93 (45)	0.094
Etiology				0.639
M. avium	324 (51)	222 (52)	102 (50)	
M. intracellulare	307 (49)	204 (48)	103 (50)	
Radiological form				0.033
Nodular bronchiectatic form	520 (82)	341 (80)	179 (87)	
Fibrocavitary form	111 (18)	85 (20)	26 (13)	

a Data are presented as number (percentage). BACES, body mass index <18.5 kg/m^2^, age ≥65 years, cavity, erythrocyte sedimentation rate (men >15 mm/h, women >20 mm/h), and sex (male).

b Includes current and former smokers.

Throughout the study period with a median of 36.0 months (interquartile range [IQR] 24.0 to 53.0 months), 205 (32%) patients experienced redevelopment. Of the 205 patients who experienced redevelopment, 118 (58%) had relapse, and 87 (42%) had reinfection. Among the 87 patients with reinfection, the most common etiology was M. intracellulare (*n* = 21), followed by M. avium (*n* = 20) (Table S1). Patients who experienced redevelopment were less likely to have a cavitary lesion than were patients who did not experience redevelopment (29% versus 38%, *P* = 0.033). In addition, the proportion of patients who had NB was significantly higher in the redevelopment group than in the nonredevelopment group (87% versus 80%, *P* < 0.001).

### Comparisons of treatment periods according to redevelopment status.

The treatment periods of MAC-PD patients according to redevelopment status are shown in Table 2. For this analysis, the treatment period was divided into so-called the initial phase, which was the time between the initiation of antibiotics and culture conversion, and the maintenance period. The initial phase did not differ between the two groups.

**TABLE 2:** Comparisons of treatment period according to redevelopment of MAC-PD^*a*^

Characteristics	Total (*n* = 631)	Without redevelopment (*n* = 426)	With redevelopment (*n* = 205)	*P* value
Time between initiation of antibiotics and culture conversion, mo	1.0 (1.0–3.0)	2.0 (1.0–4.0)	1.0 (1.0–3.0)	0.078
Maintenance period, mo^*b*^	14.0 (12.0–19.0)	14.0 (12.0–17.0)	15.0 (13.0–22.0)	<0.001
Maintenance period				
≤12 mo	87 (14)	67 (16)	20 (10)	0.056
Maintenance period of ≤12 mo group, mo	10.0 (8.5–12.0)	10.0 (8.0–11.0)	11.0 (9.0–12.0)	0.168
>12 mo	544 (86)	359 (84)	185 (90)	0.056
Maintenance period of >12 mo group, mo	15.0 (13.0–21.0)	15.0 (13.0–18.0)	15.0 (14.0–22.0)	0.003
Overall treatment duration, mo	18.0 (15.0–24.0)	17.0 (15.0–23.0)	18.0 (15.0–24.0)	0.001
Time from treatment completion to redevelopment, mo	NA	NA	18.0 (7.0–34.0)	
Total follow-up duration, mo	36.0 (24.0–53.0)	35.0 (23.0–52.0)	38.0 (29.0–53.0)	0.008

a Data are presented as number (percentage) or median (interquartile range). NA, not applicable.

b Time from culture conversion to treatment completion.

The median maintenance periods in the redevelopment and nonredevelopment groups were 15.0 months (IQR 13.0 to 22.0 months) and 14.0 months (IQR 12.0–17.0 months), respectively, with the maintenance period being significantly longer in the redevelopment group (*P* < 0.001). When patients were divided into subgroups based on the 12-month maintenance period, the redevelopment status was comparable between the ≤12-month (*n* = 87) and >12-month (*n* = 544) groups. Among the 87 patients with a maintenance period ≤12 months, the median maintenance periods were 11.0 (IQR 9.0–12.0) months and 10.0 (IQR, 8.0–11.0) months in the redevelopment and nonredevelopment groups, respectively (*P* = 0.168). Among the 544 patients with a maintenance period >12 months, the redevelopment group tended to have a wider range for the maintenance period, with 15.0 months (IQR 14.0 to 22.0 months) in the redevelopment versus 15.0 months (IQR, 13.0 to 18.0 months) in the nonredevelopment group (*P* = 0.003).

**FIG 1:**
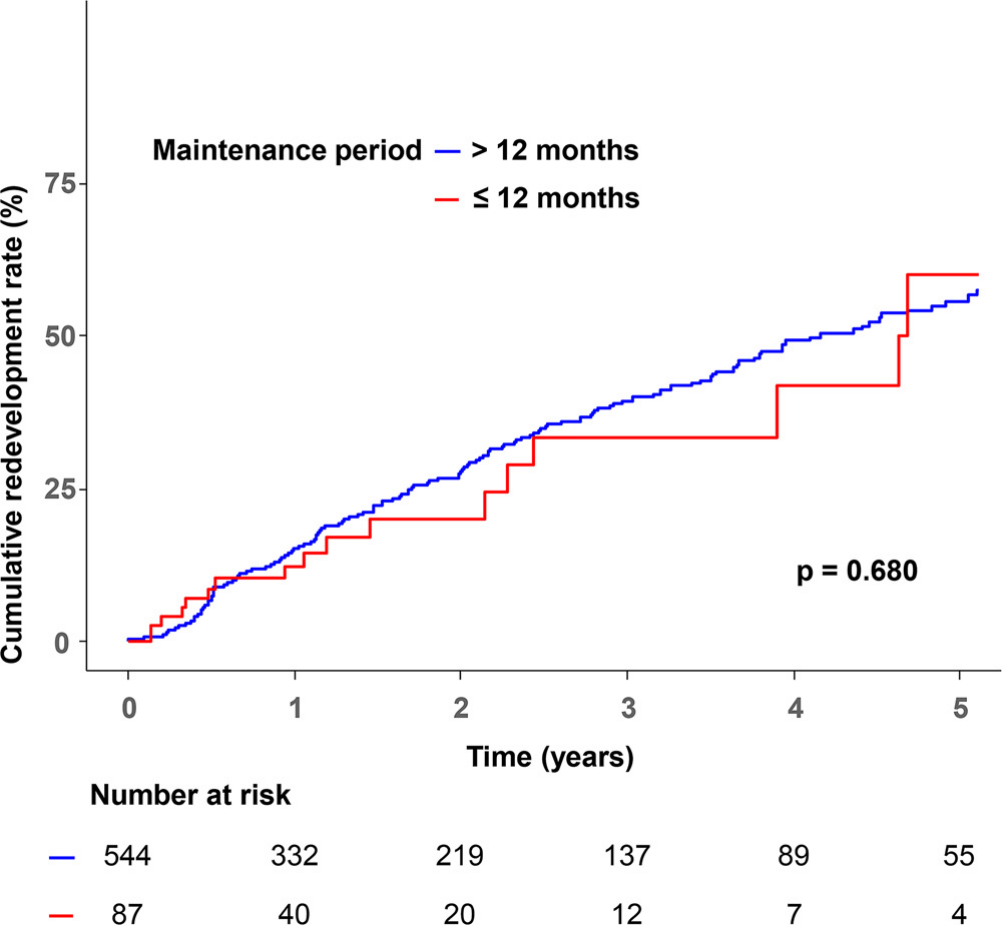
Cumulative rate of redevelopment according to the length of the maintenance period. The maintenance period was divided into two groups, ≤12 months and >12 months.

The cumulative redevelopment rate did not significantly differ according to the 12-month maintenance period (Fig. 1, Kaplan-Meier analysis; log-rank test *P* = 0.680). However, the overall treatment duration was longer in the redevelopment group than in the nonredevelopment group (Table 2). The median time from treatment completion to redevelopment among patients who experienced redevelopment was 18.0 months (IQR 7.0–34.0 months).

### Redevelopment according to the maintenance period in the BACES severity groups.

Patients were categorized into one of three severity groups (mild, moderate, or severe) according to the BACES score (Table 3). Patients with a higher BACES severity score were more likely to have underlying pulmonary disease, a smoking history, and greater proportions of M. intracellulare and the FC form. While the time from the initiation of antibiotics to culture conversion increased with higher severity, the maintenance periods were similar between three groups. Also, no significant difference in the rate of redevelopment was observed among three groups.

**TABLE 3:** Characteristics of study patients at the time of diagnosis with MAC-PD according to BACES severity^*a*^

Characteristics	Mild (BACES 0,1) (*n* = 262)	Moderate (BACES 2,3) (*n* = 315)	Severe (BACES 4,5) (*n* = 54)	*P* value
Comorbidity				
Previous pulmonary tuberculosis	69 (26)	128 (41)	28 (52)	<0.001
Chronic obstructive pulmonary disease	19 (7)	42 (13)	12 (22)	0.003
Chronic pulmonary aspergillosis	0 (0)	5 (2)	7 (13)	<0.001
Lung cancer	4 (2)	5 (2)	4 (7)	0.015
Ever-smoker^*b*^	20 (8)	95 (30)	37 (69)	<0.001
Acid-fast bacillus smear positivity	77 (29)	145 (46)	33 (61)	<0.001
Etiology				<0.001
M. avium	160 (61)	152 (48)	12 (22)	
M. intracellulare	102 (39)	163 (52)	42 (78)	
Radiological form				<0.001
Nodular bronchiectatic form	258 (98)	242 (77)	20 (37)	
Fibrocavitary form	4 (2)	73 (23)	34 (63)	
Time between initiation of antibiotics and culture conversion, mo	1.0 (1.0–3.0)	2.0 (1.0–4.0)	3.0 (2.0–5.0)	<0.001
Maintenance period, mo^*c*^	14.0 (12.0–17.0)	14.0 (12.0–20.5)	15.0 (12.0–21.0)	0.394
Overall treatment duration, mo	16.0 (15.0–21.0)	18.0 (15.0–24.0)	19.0 (15.0–24.0)	<0.001
Total follow-up duration, mo	37.5 (24.0–56.0)	36.0 (24.0–51.5)	31.5 (25.0–51.0)	0.469
Redevelopment	94 (36)	100 (32)	11 (20)	0.079

a Data are presented as number (percentage), or median (interquartile range). MAC-PD: Mycobacterium avium complex-pulmonary disease; BACES, body mass index <18.5 kg/m^2^, age ≥65 years, cavity, erythrocyte sedimentation rate (men >15 mm/h, women >20 mm/h), and sex (male).

b Includes current and former smokers.

c Time from culture conversion to treatment completion.

**FIG 2:**
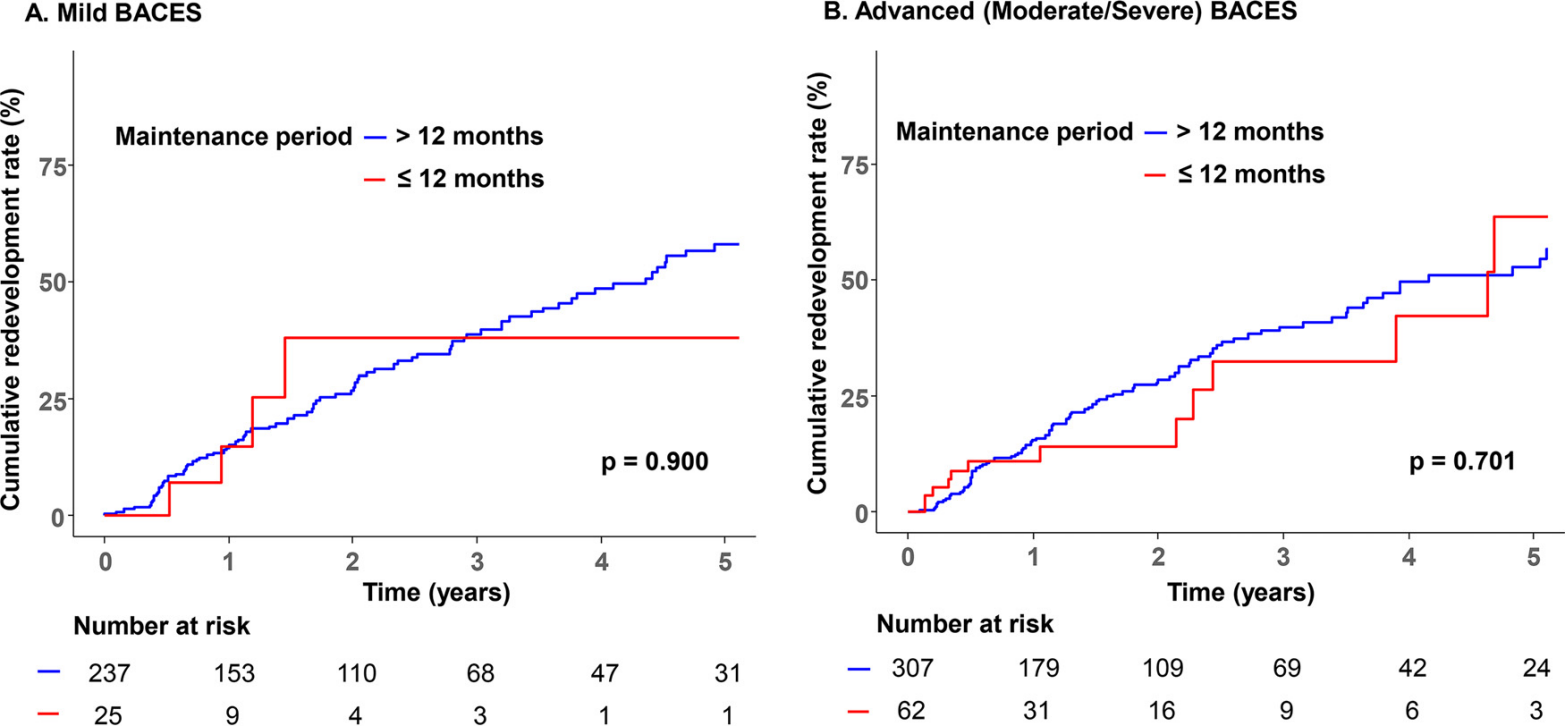
Cumulative rate of redevelopment of each BACES group, according to the maintenance period. (A) Mild BACES; (B) advanced (moderate/severe) BACES. BACES, body mass index, age, cavity, erythrocyte sedimentation rate, and sex.

The BACES severity groups were regrouped as mild (BACES mild, *n* = 262) and advanced disease (BACES moderate or severe, *n* = 369), and the cumulative rate of redevelopment according to the 12-month maintenance period was evaluated (Fig. 2). In the mild disease group, the cumulative rate of redevelopment according to the 12-month maintenance period did not significantly differ between subgroups (Fig. 2A, Kaplan-Meier analysis, log-rank test *P* = 0.900), similar to the findings in the advanced group (Fig. 2B, log-rank test *P* = 0.701).

In addition, analysis according to the radiological phenotype and etiology showed comparable results in terms of redevelopment (Tables S2 and S3). The cumulative rate of redevelopment according to the radiological phenotype or etiology did not significantly differ between subgroups (Fig. S1 and S2), results that were consistent with the findings of additional analyses according to the 12-month maintenance period (Fig. S3 and S4).

### Risk factors related to redevelopment.

Univariate and multivariate analyses were performed with various combinations of factors associated with redevelopment (Tables S4-1, S4-2, and S4-3), and the effects of a >12-month maintenance period on redevelopment were summarized (Table 4). Three multivariate models were constructed: *model 1* (Table S4-1), adjusted for BACES severity, acid-fast bacillus smear positivity, underlying disease, smoking history, etiology, and maintenance period; *model 2* (Table S4-2), adjusted for body mass index (<18.5 kg/m^2^), age (≥65 years), cavity, erythrocyte sedimentation rate >15 mm/h in men and >20 mm/h in women, and sex (male) instead of the BACES in *model 1*; and *model 3* (Table S4-3), adjusted for phenotype instead of the “cavity” factor used in *model 2*. Multivariate analyses using model 1 and model 2 did not demonstrate any factors that were significantly associated with an elevated risk of redevelopment. In contrast, the NB form in *model 3* was positively associated with redevelopment (hazard ratio, 1.61; 95% confidence interval, 1.01 to 2.57).

**TABLE 4:** Effects of maintenance period on redevelopment of MAC-PD^*a*^

HR of Maintenance period >12 mo^*b*^	HR (95% CI, *P* value)
Crude HR	0.99 (0.63–1.58, 0.976)
Model 1^*c*^	0.99 (0.61–1.59, 0.961)
Model 2^*d*^	1.05 (0.65–1.71, 0.838)
Model 3^*e*^	1.05 (0.65–1.71, 0.839)

a HR, hazard ratio; CI, confidence interval. Detailed variables in each model are described in Table S3.

b Time from culture conversion to treatment completion.

c *Model 1*: adjusted for BACES severity, acid-fast bacillus smear positivity, underlying disease, smoking, and etiology.

d *Model 2*: adjusted for body mass index (<18.5 kg/m^2^), age (≥65 years), cavity, erythrocyte sedimentation rate (>15 mm/h in male and >20 mm/h in female), and sex (male), instead of ‘BACES’ in *model 1.*

e *Model 3*: adjusted for phenotype instead of the cavity factor used in *model 2.*

## DISCUSSION

To our knowledge, this is the largest study to evaluate the redevelopment of NTM-PD after achievement of microbiological cure in MAC-PD patients, and we assessed the impact of the antibiotic maintenance period according to various clinical factors. A greater proportion of NB form in the redevelopment group was the only difference between the redevelopment group and nonredevelopment group. In addition, no significant difference in the rate of redevelopment was observed between patients with maintenance period >12 months and patients with maintenance period ≤12 months. This result was consistent with the findings of further analyses that considered disease severity using the BACES system.

Our observation that the rate of redevelopment did not differ according to the maintenance period was notable, especially given that prolonging the maintenance period beyond 12 months did not decrease the rate of redevelopment, which suggests that a certain subset of NTM-PD patients eventually experience redevelopment after specific interval, regardless of maintenance period extension. This result can be explained by reinfection, rather than relapse of the initial causative species. Several studies have demonstrated that new MAC-positive sputum cultures after initial culture conversion are usually caused by reinfection, rather than disease relapse (10, 19, 20). These findings reflect the multifactorial nature of NTM-PD and that its development is highly affected by patient susceptibility to NTM-PD and unavoidable environmental exposure to NTM.

We also analyzed the impact of the maintenance period on redevelopment while considering disease severity, as determined by the BACES score. The BACES score, first proposed by Kim et al. in 2020 (18), was developed to predict mortality in patients with NTM-PD. The estimated 5-year risks of mortality are 1.2% with a BACES score of 0 and 82.9% with a BACES score of 5. In our study, moderate to severe BACES score group was treated for a longer overall antibiotic period. However, the maintenance period was similar regardless of the BACES score, and the redevelopment rate did not differ between three groups. These results suggest that, although higher BACES score group needed more antibiotic therapy to achieve a negative conversion, the redevelopment rate may not have been affected by the length of the maintenance period, suggesting the need to consider the high proportion of reinfections in the redevelopment group.

Additional analysis sorted according to phenotype revealed that patients with the NB form had higher redevelopment rates. A previous study showed a higher cumulative rate of redevelopment was observed in patients with the NB form (33%) than the FC form (16%), suggesting the clinical phenotype as a risk factor for redevelopment (10). Another study by Lee et al. retrospectively analyzed the risk factors for recurrence of MAC-PD and showed that only the NB form was independently associated with an increased risk of recurrence (11). The higher redevelopment rate in the NB form has been explained by repeated infection with multiple strains, which is related to patient host susceptibility (21, 22). Based on these NTM-PD traits, there is a need to establish an individualized treatment duration that considers host factors, and this approach may be more effective than the establishment of a comprehensive antibiotic duration.

Our multivariable models showed a positive association between the NB form and redevelopment. However, the slope of the cumulative rate of redevelopment in each phenotype must be considered (Fig. S1). The steeper slope of the FC form in the first 1 to 2 years of follow-up compared to the NB form suggests that a higher proportion of patients with the FC form experience redevelopment within a short period, while redevelopment occurs more slowly in the NB form. Therefore, the long follow-up period in our study may have had the effect of further emphasizing that the NB form is a risk factor for redevelopment.

Our study has several limitations. First, this was a retrospective study from a single referral center, which may limit the generalizability. Second, because we did not perform a genotypic analysis, we could not determine whether redevelopment was caused by strains with different genotypes in reinfection group. Third, because patients who discontinued antibiotics early in the treatment period were excluded, there is a possibility of underestimation of the redevelopment rate. Fourth, the clinical situations of each patient at 12 months after culture conversion and the reasons for prolongation of duration of antibiotics were not assessed. Thus, the decision by clinicians may have led to selection bias of study patients. Lastly, BACES has not been validated in many patient cohorts, and further studies are required to investigate the host factors that are related to redevelopment and the optimal duration of the maintenance period.

In conclusion, no other factors associated with redevelopment were identified, except for the possibility of a higher redevelopment rate in the NB form of MAC-PD. Extending the antibiotic maintenance period more than 12 months was not associated with the reducing redevelopment rate despite adjustment for disease severity, suggesting the need to further optimize the duration of the antibiotic maintenance period.

## MATERIALS AND METHODS

### Study design and population.

We screened consecutive patients who were newly diagnosed with MAC-PD between April 1994 and March 2021 from the NTM Registry of Samsung Medical Center, a referral hospital in Seoul, South Korea. From April 1994 to December 2007, data were obtained from a retrospective cohort, and beginning in January 2008, data were obtained from an ongoing Institutional Review Board-approved prospective observational cohort (ClinicalTrials.gov identifier: NCT00970801). In total, 1,415 patients who started antibiotics without adjuvant surgery were identified. Of them, patients without available data for BACES severity (*n* = 272), treatment outcome (*n* = 304), or treatment failure/death (*n* = 208) were excluded from the analysis. Finally, 631 MAC-PD patients who achieved microbiological cure through antibiotic therapy were included in this study (Fig. S5). Clinical outcome data were last updated in May 2021.

### Microbiological and radiological examinations.

The treatment strategy was based on macrolide with rifamycin and ethambutol, according to the official guidelines, in all patients (5, 6). Patients who achieved microbiological cure for MAC-PD were followed up at 3–6-month intervals in an outpatient clinic, and sputum and radiological examinations were performed at each visit. Sputum acid-fast bacillus smears and cultures were performed using standard methods. Specimens were cultured on 3% Ogawa solid medium (Shinyang, Seoul, South Korea) and in liquid broth medium in mycobacterial growth indicator tubes (Becton, Dickinson and Co., Sparks, MD, USA). PCR-restriction fragment length polymorphism analysis or reverse-blot hybridization of the *rpoB* gene were used to identify the NTM species. Beginning in June 2014, NTM species were identified using nested multiplex PCR and a reverse-hybridization assay of the internal transcribed spacer region (AdvanSure Mycobacteria GenoBlot Assay; LG Life Sciences, Seoul, South Korea). The radiological form of the patients was categorized as FC or NB form.

### BACES severity.

The severity of MAC-PD was calculated using the BACES score: body mass index <18.5 kg/m^2^; age ≥65 years; presence of cavity; elevated erythrocyte sedimentation rate (men >15 mm/h, women >20 mm/h); and male sex; each one point (18). All patients were classified into the following three groups according to their severity score: mild (0 to 1 point), moderate (2 to 3 points), or severe (4 to 5 points).

### Treatment outcome measurement.

Treatment outcomes were assessed based on the modified NTM-NET consensus statement (23) because a genotype analysis was not included in the present study. Relapse was defined as the emergence of at least two positive cultures with the same NTM species encountered during the initial treatment, while reinfection was defined as the emergence of a different NTM species from sputum samples during the follow-up period. Redevelopment included both relapse and reinfection.

Sputum negative culture conversion was defined as at least three consecutive negative sputum cultures, collected at least 4 weeks apart. The time of conversion was defined as the date of the first negative culture. In this study, the “maintenance period” was defined as the time interval between culture conversion and the completion of MAC-PD treatment.

### Statistical analyses.

All data are presented as number (percentages) for categorical variables and median (IQR) for continuous variables. Continuous variables were compared using the Student's *t* test or the Mann-Whitney test, and categorical variables were compared using the χ^2^ or Fisher’s exact test. The Kaplan-Meier method was used to estimate the cumulative redevelopment rates of MAC-PD patients, and the log-rank test was used to compare the survival curves. Factors associated with redevelopment in MAC-PD patients were analyzed using the multivariate Cox proportional-hazards regression model. Statistical analyses were performed using R software (version 4.1.0; R Development Core Team, Vienna, Austria).
