# Supplementary material for: Antibiotic Maintenance and Redevelopment of Nontuberculous Mycobacteria Pulmonary Disease after Treatment of Mycobacterium avium Complex Pulmonary Disease
Source: Microbiol Spectr. 2022 Aug 11;10(4):e01088-22. doi: 10.1128/spectrum.01088-22 (PMC9431257; doi:10.1128/spectrum.01088-22)

Supplementary material

Antibiotic Maintenance and Redevelopment of *Mycobacterium avium* Complex Pulmonary Disease

Sungmin Zo[1], Hojoong Kim[1], O Jung Kwon[1], Byung Woo Jhun[1]

[1]Division of Pulmonary and Critical Care Medicine, Department of Medicine, Samsung Medical Center, Sungkyunkwan University School of Medicine, Seoul, Republic of Korea.

Correspondence: Byung Woo Jhun, Division of Pulmonary and Critical Care Medicine, Department of Medicine, Samsung Medical Center, Sungkyunkwan University School of Medicine, 81 Irwon-ro, Gangnam-gu, Seoul 06351, Republic of Korea. E-mail: byungwoo.jhun@gmail.com

Table S1 Etiology of reinfection

Original etiology	Etiology of reinfection							
	M. avium	*M. intracellulare*	*M. abscessus*	*M. massiliense*	*M. abscessus complex*	*M. kansasii*	Mixed	Etc.*
M. avium (n=45)	-	21	10	6	-	-	6	2
M. intracellulare (n=42)	20	-	7	2	1	2	10	-

Etc.* included *M. fortuitum complex* and *M. shimoidei.*

Table S2 Characteristics of study patients at the time of diagnosis with MAC-PD according to phenotype

Characteristics	Nodular bronchiectatic (n=520)	Fibrocavitary (n=111)	p-value
BACES			
Body mass index <18.5 kg/m^2	113 (22)	38 (34)	0.007
Age ≥65 years	92 (18)	31 (28)	0.019
Cavity	113 (22)	107 (96)	<0.001
Elevated erythrocyte sedimentation rate	361 (69)	92 (83)	0.006
Sex, male	135 (26)	72 (65)	<0.001
BACES severity			<0.001
Mild	258 (50)	4 (4)	
Moderate	242 (46)	73 (66)	
Severe	20 (4)	34 (31)	
Comorbidity			
Previous pulmonary tuberculosis	159 (31)	66 (60)	<0.001
Chronic obstructive pulmonary disease	47 (9)	26 (23)	<0.001
Chronic pulmonary aspergillosis	3 (1)	9 (8)	<0.001
Lung cancer	5 (1)	8 (7)	<0.001
Ever-smoker*	94 (18)	58 (52)	<0.001
Acid-fast bacillus smear positivity	174 (34)	81 (73)	<0.001
Etiology			0.005
M. avium	281 (54)	43 (39)	
M. intracellulare	239 (46)	68 (61)	
Time between initiation of antibiotics and culture	1.0 (1.0-3.0)	3.0 (1.0-5.0)	<0.001

conversion, months			
Maintenance period[‡], months	14.0 (12.0-17.5)	17.0 (13.0-22.0)	<0.001
Overall treatment duration, months	17.0 (15.0-23.0)	23.0 (16.0-24.0)	<0.001
Total follow-up duration, months	35.0 (24.0-51.0)	38.0 (24.5-62.0)	0.193
Redevelopment	179 (34)	26 (23)	0.033

Data are presented as number (%) or median (interquartile range). MAC-PD, *Mycobacterium avium* complex-pulmonary disease; BACES, body mass index <18.5kg/m^2, age ≥65 years, cavity, erythrocyte sedimentation rate (men >15 mm/h, women >20 mm/h), and sex (male). [*]Includes current and former smokers. [‡]Time from culture conversion to treatment completion.

Table S3 Characteristics of study patients at the time of diagnosis with MAC-PD according to etiology

Characteristics	*M. avium* (n=324)	*M. intracellulare* (n=307)	p-value
BACES			
Body mass index <18.5 kg/m^2	61 (19)	90 (29)	0.003
Age ≥65 years	40 (12)	83 (27)	<0.001
Cavity	95 (29)	125 (41)	0.004
Elevated erythrocyte sedimentation rate	216 (67)	237 (77)	0.004
Sex, male	97 (30)	110 (36)	0.136
BACES severity			<0.001
Mild	160 (49)	102 (33)	
Moderate	152 (47)	163 (53)	
Severe	12 (4)	42 (14)	
Comorbidity			
Previous pulmonary tuberculosis	104 (32)	121 (39)	0.067
Chronic obstructive pulmonary disease	31 (10)	42 (14)	0.136
Chronic pulmonary aspergillosis	5 (2)	7 (2)	0.700
Lung cancer	4 (1)	9 (3)	0.223
Ever-smoker*	64 (20)	88 (29)	0.012
Acid-fast bacillus smear positivity	121 (37)	134 (44)	0.126
Radiological form			0.005
Nodular bronchiectatic form	281 (87)	239 (78)	
Fibrocavitary form	43 (13)	68 (22)	
Time between initiation of antibiotics and culture conversion, months	1.0 (1.0-3.0)	2.0 (1.0-4.0)	0.206

Maintenance period[‡], months	14.0 (12.0-18.0)	14.0 (12.0-20.0)	0.974
Overall treatment duration, months	18.0 (15.0-24.0)	18.0 (15.0-24.0)	0.226
Total follow-up duration, months	38.0 (25.0-57.0)	34.0 (24.0-50.5)	0.105
Redevelopment	102 (32)	103 (34)	0.639

Data are presented as number (%) or median (interquartile range). MAC-PD, *Mycobacterium avium* complex-pulmonary disease; BACES, body mass index <18.5kg/m^2, age ≥65 years, cavity, erythrocyte sedimentation rate (men >15 mm/h, women >20 mm/h), and sex (male). [*]Includes current and former smokers. [‡]Time from culture conversion to treatment completion.

Figure S1 Cumulative rate of redevelopment of MAC-PD according to phenotype (NB, nodular bronchiectatic; FC, fibrocavitary).

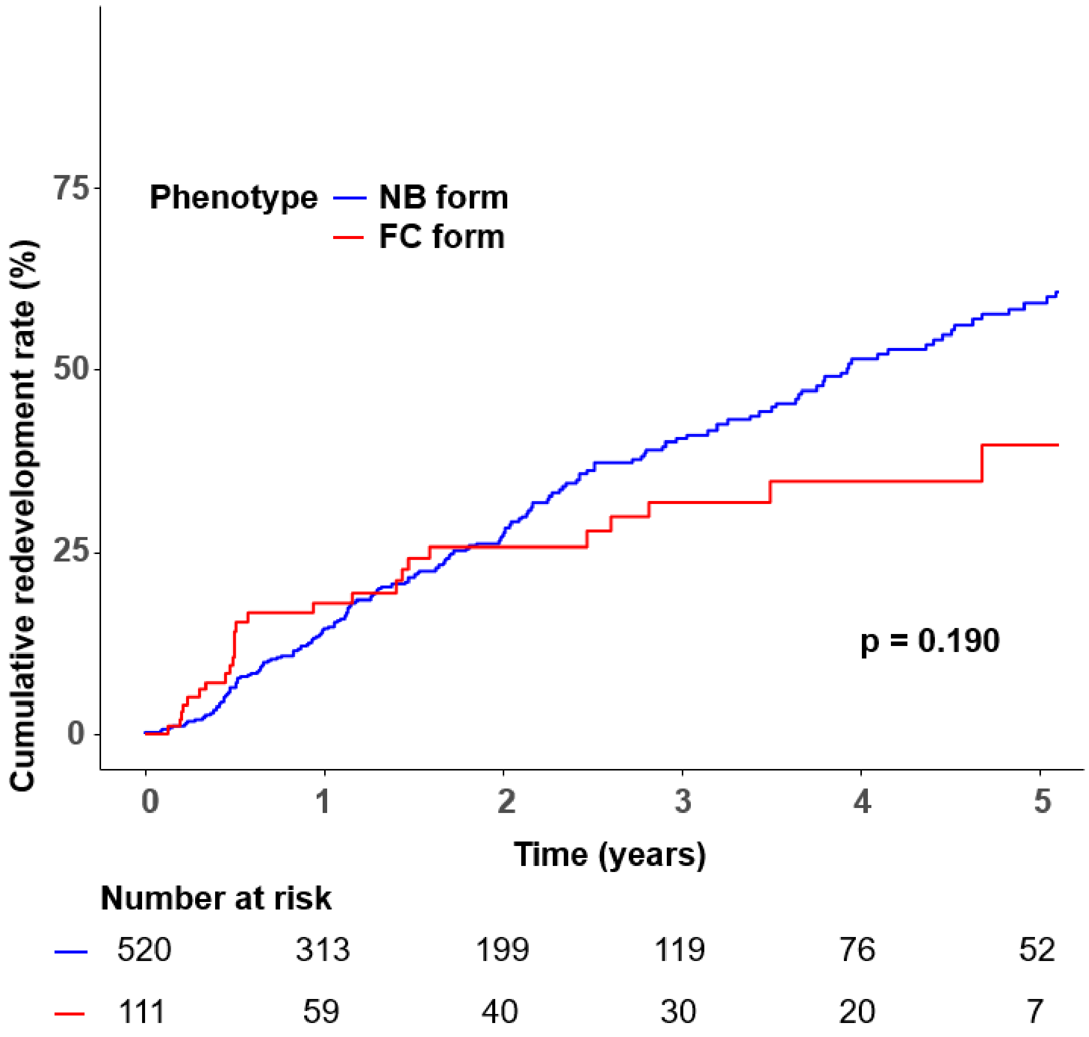

Figure S2 Cumulative rate of redevelopment of MAC-PD according to etiology.

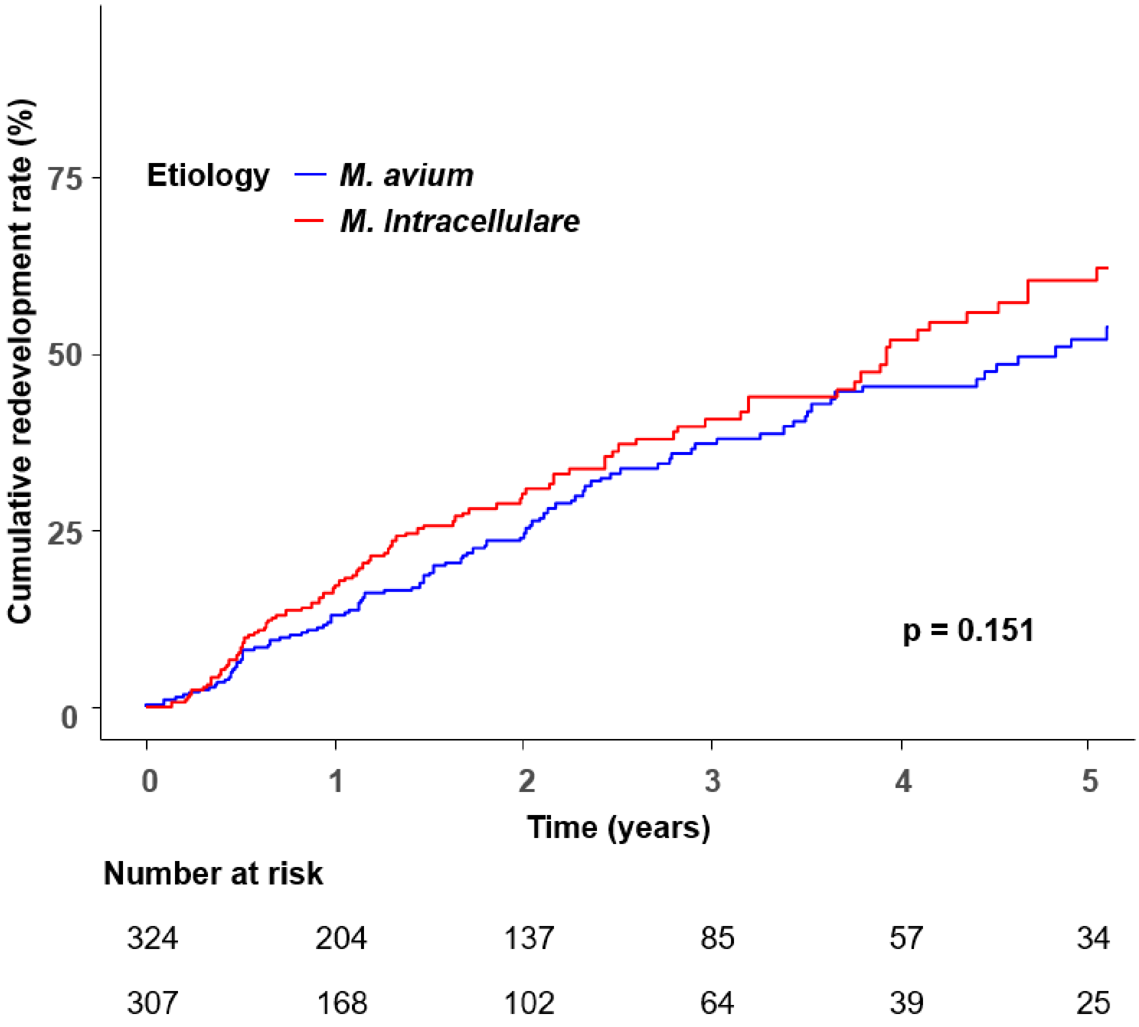

Figure S3 Cumulative rate of redevelopment of each phenotype of MAC-PD, according to maintenance period (NB, nodular bronchiectatic; FC, fibrocavitary).

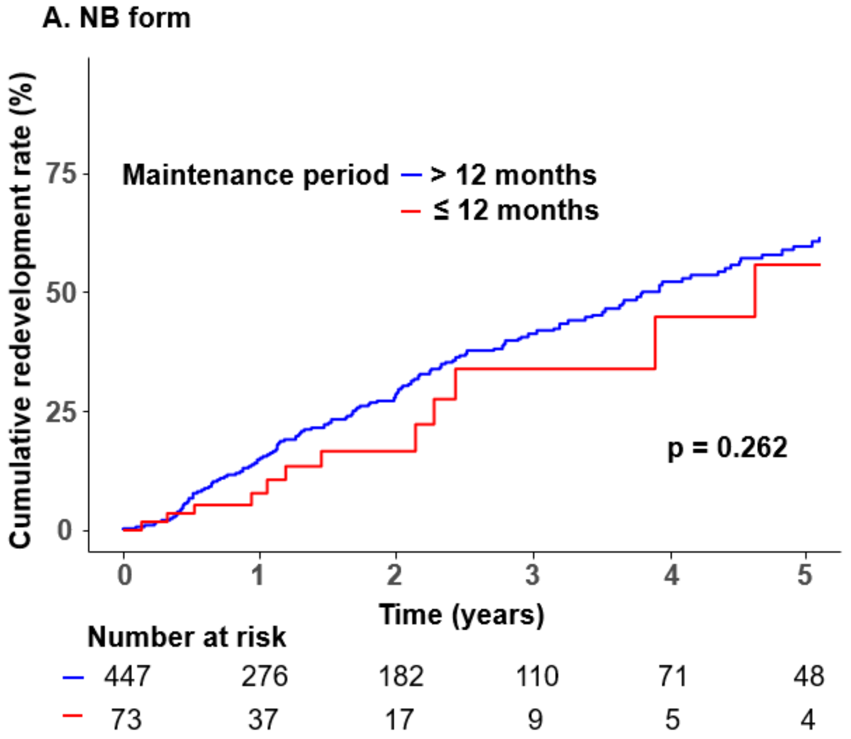

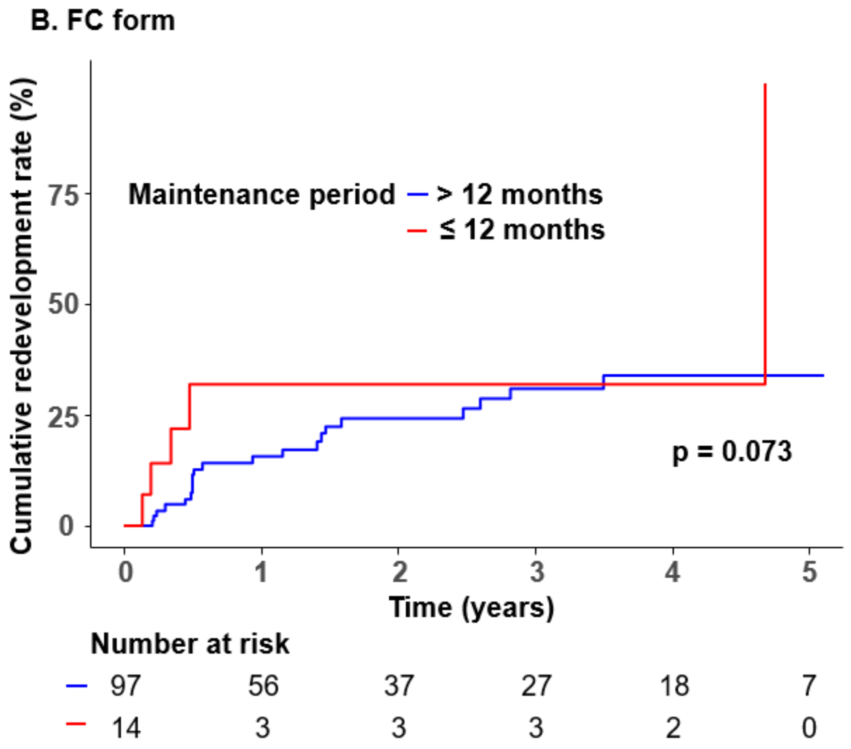

Figure S4 Cumulative rate of redevelopment of MAC-PD according to etiology.

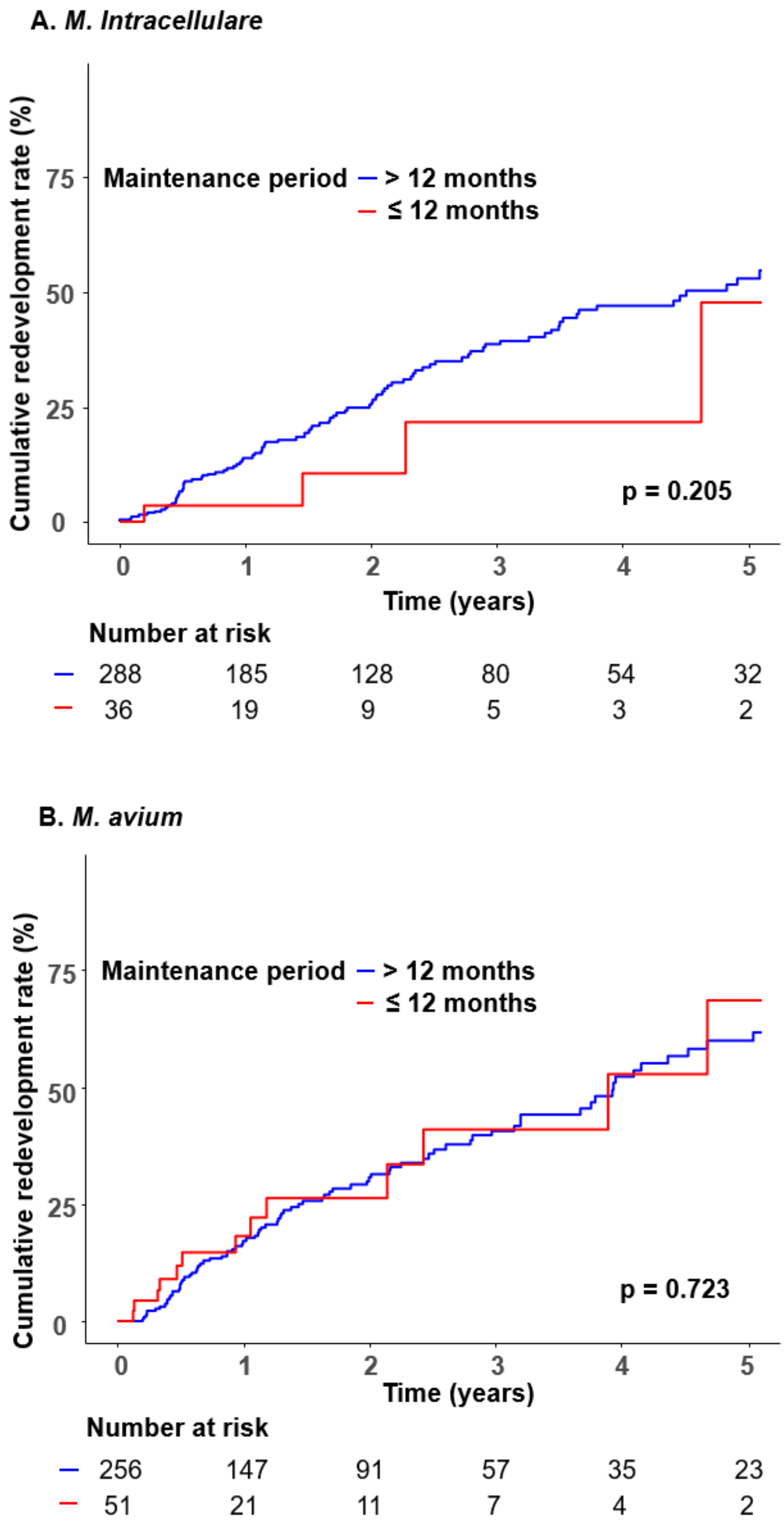

Table S4-1 Risk factors related to redevelopment of MAC-PD in 631 study patients (*Model 1*)

Characteristics	Univariate analysis		Multivariate analysis	
	HR (95% CI)	p-value	HR (95% CI)	p-value
BACES				
Mild	reference		reference	
Moderate	1.06 (0.80-1.41)	0.686	0.97 (0.71-1.31)	0.832
Severe	0.77 (0.41-1.44)	0.416	0.63 (0.30-1.31)	0.216
Acid-fast bacillus smear positivity	1.09 (0.83-1.44)	0.533	1.08 (0.81-1.44)	0.594
Previous pulmonary tuberculosis	1.09 (0.82-1.44)	0.549	1.09 (0.81-1.47)	0.562
Chronic obstructive pulmonary disease	0.94 (0.61-1.43)	0.765	0.89 (0.57-1.37)	0.595
Chronic pulmonary aspergillosis	0.59 (0.19-1.85)	0.363	0.70 (0.20-2.42)	0.575
Lung cancer	1.03 (0.38-2.78)	0.950	1.06 (0.39-2.88)	0.908
Ever-smoker*	1.20 (0.87-1.66)	0.256	1.28 (0.90-1.82)	0.162
Etiology				
M. avium	reference		reference	
M. intracellulare	1.24 (0.94-1.63)	0.124	1.26 (0.95-1.67)	0.112
Maintenance period‡				
≤12 months	reference		reference	
>12 months	0.99 (0.63-1.58)	0.976	0.99 (0.61-1.59)	0.961

BACES, body mass index <18.5kg/m^2, age ≥65 years, cavity, erythrocyte sedimentation rate (men >15 mm/h, women >20 mm/h), and sex (male). *Includes current and former smokers. ‡Time from culture conversion to treatment completion

Table S4-2 Risk factors related to redevelopment of MAC-PD in 631 study patients (*Model 2*)

Characteristics	Univariate analysis		Multivariate analysis	
	HR (95% CI)	p-value	HR (95% CI)	p-value
Body mass index <18.5 kg/m^2	1.05 (0.77-1.43)	0.769	1.08 (0.76-1.52)	0.678
Age ≥65 years	1.12 (0.77-1.64)	0.552	0.98 (0.66-1.48)	0.940
Cavity	0.86 (0.64-1.16)	0.331	0.78 (0.55-1.10)	0.153
Elevated erythrocyte sedimentation rate*	1.00 (0.74-1.34)	0.977	1.03 (0.75-1.41)	0.860
Sex, male	1.19 (0.89-1.60)	0.239	1.21 (0.74-1.99)	0.445
Acid-fast bacillus smear positivity	1.09 (0.83-1.44)	0.533	1.12 (0.83-1.51)	0.446
Previous pulmonary tuberculosis	1.09 (0.82-1.44)	0.549	1.10 (0.82-1.49)	0.520
Chronic obstructive pulmonary disease	0.94 (0.61-1.43)	0.765	0.88 (0.57-1.37)	0.569
Chronic pulmonary aspergillosis	0.59 (0.19-1.85)	0.363	0.53 (0.16-1.77)	0.300
Lung cancer	1.03 (0.38-2.78)	0.950	1.03 (0.38-2.78)	0.959
Ever-smoker‡	1.20 (0.87-1.66)	0.256	1.06 (0.62-1.80)	0.835
Etiology				
M. avium	reference		reference	
M. intracellulare	1.24 (0.94-1.63)	0.124	1.24 (0.93-1.66)	0.135
Maintenance period¶				
≤12 months	reference		reference	
>12 months	0.99 (0.63-1.58)	0.976	1.05 (0.65-1.71)	0.838

*Men >15 mm/h, women >20 mm/h. ‡Includes current and former smokers. ¶Time from culture conversion to treatment completion.

Table S4-3 Risk factors related to redevelopment of MAC-PD in 631 study patients (*Model 3*)

Characteristics	Univariate analysis		Multivariate analysis	
	HR (95% CI)	p-value	HR (95% CI)	p-value
Body mass index <18.5 kg/m^2	1.05 (0.77-1.43)	0.769	1.10 (0.78-1.55)	0.594
Age ≥65 years	1.12 (0.77-1.64)	0.552	1.00 (0.67-1.51)	0.983
Elevated erythrocyte sedimentation rate*	1.00 (0.74-1.34)	0.977	1.01 (0.74-1.37)	0.960
Sex, male	1.19 (0.89-1.60)	0.239	1.22 (0.74-2.00)	0.428
Acid-fast bacillus smear positivity	1.09 (0.83-1.44)	0.533	1.13 (0.84-1.51)	0.417
Previous pulmonary tuberculosis	1.09 (0.82-1.44)	0.549	1.11 (0.82-1.49)	0.496
Chronic obstructive pulmonary disease	0.94 (0.61-1.43)	0.765	0.91 (0.58-1.41)	0.672
Chronic pulmonary aspergillosis	0.59 (0.19-1.85)	0.363	0.64 (0.18-2.20)	0.476
Lung cancer	1.03 (0.38-2.78)	0.950	1.14 (0.42-3.12)	0.795
Ever-smoker‡	1.20 (0.87-1.66)	0.256	1.10 (0.65-1.88)	0.718
Phenotype				
Fibrocavitary form	reference		reference	
Nodular bronchiectatic form	1.39 (0.92-2.09)	0.120	1.61 (1.01-2.57)	0.047
Etiology				
M. avium	reference		reference	
M. intracellulare	1.24 (0.94-1.63)	0.124	1.22 (0.92-1.63)	0.173
Maintenance period¶				
≤12 months	reference		reference	
>12 months	0.99 (0.63-1.58)	0.976	1.05 (0.65-1.71)	0.839

[*]Men >15 mm/h, women >20 mm/h. [‡]Includes current and former smokers. [¶]Time from culture conversion to treatment completion.

Figure S5 Study patients. MAC-PD, *Mycobacterium avium* complex-pulmonary disease; BACES, body mass index, age, cavity, erythrocyte sedimentation rate, and sex.

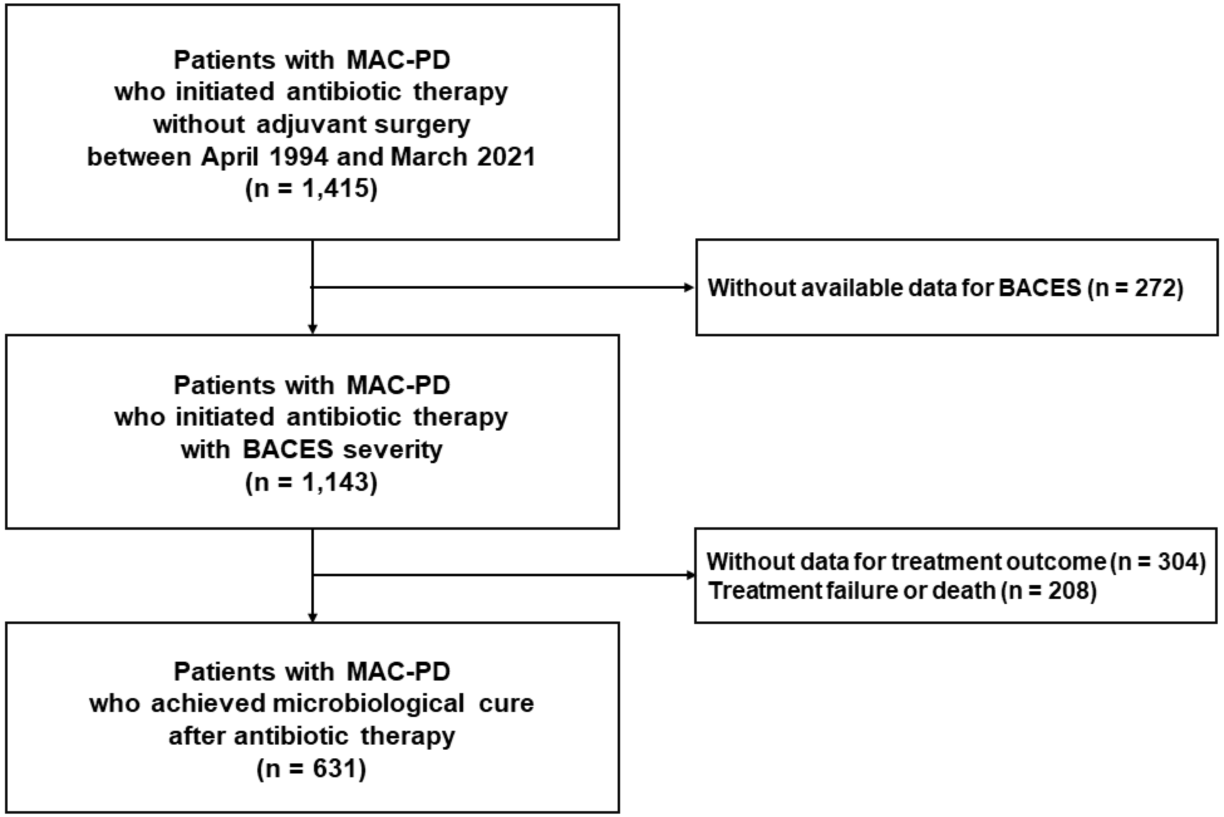

Supplement: Supplemental file 1 — Supplemental material. Download spectrum.01088-22-s0001.pdf, PDF file, 0.6 MB [file spectrum.01088-22-s0001.pdf]
